# Ranges of phenotypic flexibility in healthy subjects

**DOI:** 10.1186/s12263-017-0589-8

**Published:** 2017-12-06

**Authors:** T. J. van den Broek, G. C. M. Bakker, C. M. Rubingh, S. Bijlsma, J. H. M. Stroeve, B. van Ommen, M. J. van Erk, S. Wopereis

**Affiliations:** 0000 0001 0208 7216 grid.4858.1 TNO, Utrechtseweg 48, 3704 HE Zeist, The Netherlands

**Keywords:** Metabolic health, Personalized health, Nutritional challenge, Data visualization, Challenge test

## Abstract

**Background:**

A key feature of metabolic health is the ability to adapt upon dietary perturbations. A systemic review defined an optimal nutritional challenge test, the “PhenFlex test” (PFT). Recently, it has been shown that the PFT enables the quantification of all relevant metabolic processes involved in maintaining or regaining homeostasis of metabolic health. Furthermore, it was demonstrated that quantification of PFT response was more sensitive as compared to fasting markers in demonstrating reduced phenotypic flexibility in metabolically impaired type 2 diabetes subjects.

**Methods:**

This study aims to demonstrate that quantification of PFT response can discriminate between different states of health within the healthy range of the population. Therefore, 100 healthy subjects were enrolled (50 males, 50 females) ranging in age (young, middle, old) and body fat percentage (low, medium, high), assuming variation in phenotypic flexibility. Biomarkers were selected to quantify main processes which characterize phenotypic flexibility in response to PFT: flexibility in glucose, lipid, amino acid and vitamin metabolism, and metabolic stress. Individual phenotypic flexibility was visualized using the “health space” by representing the four processes on the health space axes. By quantifying and presenting the study subjects in this space, individual phenotypic flexibility was visualized.

**Results:**

Using the “health space” visualization, differences between groups as well as within groups from the healthy range of the population can be easily and intuitively assessed. The health space showed a different adaptation to the metabolic PhenFlex test in the extremes of the recruited population; persons of young age with low to normal fat percentage had a markedly different position in the health space as compared to persons from old age with normal to high fat percentage.

**Conclusion:**

The results of the metabolic PhenFlex test in conjunction with the health space reliably assessed health on an individual basis. This quantification can be used in the future for personalized health quantification and advice.

**Electronic supplementary material:**

The online version of this article (10.1186/s12263-017-0589-8) contains supplementary material, which is available to authorized users.

## Background

In this paper, we present a comprehensive strategy with the ultimate goal of quantifying and visualizing (personal) health across a range of healthy phenotypes from the general population. We reach this goal by using a nutritional challenge test as a procedure of health assessment, coupled to a novel statistical visualization method.

A crucial aspect of health is the ability to maintain homeostasis under a large variety of continuously changing environmental conditions. Viewing health as a function of the resilience to daily stressors makes measuring phenotypic flexibility a necessary part of the quantification of health [1].

A pivotal part of the strategy presented here is the ability to assess this resilience in human subjects. Measuring the response to a (nutritional) challenge allows to quantify the metabolic ability of an individual to deal with a copious meal, and as such to assess metabolic health. Previously, it was shown that quantification of challenge-response significantly contributes to demonstrating health effects of food and nutrition in dietary intervention studies [2–4]*.* A standardized optimal nutritional challenge test was defined after the performance of a systematic literature review [5], which was named the “PhenFlex test” (PFT). Recently, this “PhenFlex test” was characterized, where 132 parameters were quantified during the 8-h response time course, that report on 26 metabolic processes distributed over seven organs (gut, liver, adipose, pancreas, vasculature, muscle, kidney) and systemic stress [6]. It showed that the adaptive capacities of the most relevant metabolic processes can be modulated by PFT. Furthermore, it was demonstrated that the PFT and defined new biomarkers are reliable in discriminating metabolically impaired subjects with type 2 diabetes from healthy subjects, since it was a more sensitive, early, and meaningful measure than the corresponding overnight fasting measure [6]. For these reasons, the quantification of the response after a (nutritional) challenge may be a good alternative for the “classic (i.e., overnight fasting) biomarkers” in nutritional and health sciences. A major remaining challenge in this field is the accurate assessment of the effect of food and nutrition on the health status of individuals, particularly in healthy subjects. Current methods used in nutritional research stem mainly from pharmaceutical and medical research, which traditionally focus on (effects from treatment on) disease, evaluating overnight fasting concentrations of certain (surrogate) biomarkers reflecting disease symptoms. This results in current nutritional science attempting to demonstrate nutritional health effects by using these surrogate or disease-related markers. Nutrition does not interact with a specific target like most drugs, but instead interacts simultaneously on a number of metabolic pathways and functions. Furthermore, the magnitude of nutritional effects is often much lower than that of commonly observed for drugs [7, 8]. Because lifestyle and nutritional interventions in many cases enhance processes that restore or maintain homeostasis, we posit that the assessment of resilience is essential for determining the impact of these interventions on health [9]. To do so, it is important to characterize the response to PFT in the healthy range of the population, ranging from an optimal towards a suboptimal response to the PFT as a measure of health.

The current study aimed to assess the ability of PFT to quantify flexibility in the healthy range of the population and whether it is possible to discriminate between optimal and suboptimal flexibility and therefore evaluated PFT response in 100 healthy male and female volunteers. We hypothesized that male and female subjects of higher age (60–70 years of age) and normal to high body fat percentage would respond differently to PFT as compared to young subjects (20–30 years of age) with a low to normal body fat percentage. Furthermore, we hypothesized that increasing adiposity (in terms of body fat percentage) would decrease phenotypic flexibility resulting in a higher metabolic age. The study is part of a larger endeavor, aiming to develop a set of standardized tools to substantiate health effects of dietary interventions [6]. Standardization of the nutritional challenge is important in generating a solid base of comparable evidence. Only with the use of a standardized challenge test, study results will be comparable and interpretable across studies. The parameters measured during the 8-h response PFT time course cover flexibility in glucose, lipid, and amino acid and vitamin metabolism as well as metabolic stress. A large section of the parameters within this selection is obtained by applying metabolic profiling, which allows for the simultaneous measurement of the challenge response for a large contingent of parameters at once and has been applied extensively in nutrition and health research [10–13]. All of the biochemical parameters measured in this study were also quantified in the previous study, allowing for the integration of both studies permitting the comparison of PFT response of the healthy range of the male population with the PFT response from 20 type 2 diabetic male subjects. To evaluate PFT responses, a statistical visualization methodology called “health space” was applied as described earlier by Bouwman et al. [14].

Concretely, we use the PFT challenge, administered to 100 healthy individuals from a range of phenotypes, together with the application of the health space modeling technique to assess and visualize phenotypic flexibility as a measure of health. The emphasis in this study lies on the visualization and comparison of phenotypic flexibility in a range of healthy phenotypes, reflecting the general population. We show that PFT challenge with the biomarker subset and health space visualization tool form a nutrition research toolbox that can be used for readily interpretable substantiation of health effects from interventions as well as for personalized health quantification and advice.

## Methods

### Subjects

The study was conducted at the Center for Human Drug Research (CHDR) in Leiden, the Netherlands. Study participants were recruited from the CHDR volunteer database and via study-specific advertisements in local media and over the internet. All subjects gave written informed consent, and the study was approved by the Independent Ethics Committee of Leiden University Medical Center (LUMC), the Netherlands. The study was conducted according to the principles of the Helsinki Declaration and in accordance with the Dutch Medical Research in Human Subjects Act (WMO). The study was performed in compliance with good clinical practice (GCP). The trial was retrospectively registered on 12 May 2017 with ID: ISRCTN10600261.

This was a single-center, explorative, open-label study among 100 healthy subjects (50 males, 50 females). All subjects were aged between 19 and 71 years and were equally assigned to ten groups based on combinations of the three phenotypic characteristics: age, body fat percentage, and gender. The groups present in the study are outlined in Table 1. Body adiposity was determined using bioelectrical impedance analysis using an Inbody 720 body composition analyzer (Biospace Co., Ltd., Korea), and subjects were grouped according to body fat percentage qualifiers (low, normal and high) as seen in the last column of Table 1. All of the groups mentioned in Table 1 contain ten individuals, for a total of 100 subjects. Phenotypic groups 1 and 6 were selected to represent “optimal phenotypic flexibility” as it is commonly perceived, while groups 5 and 10 were selected to represent “reduced phenotypic flexibility.” These will be referred to as the two reference groups. The two reference groups were used for the creation of the health space model representing the two extremes of phenotypic flexibility. The other individuals were grouped according to a combination of age and adiposity, representing a range of healthy phenotypes as commonly found in the general population. These will be referred to as “healthy range of phenotypes” and were used for evaluation of PhenFlex challenge test response and its ability to discriminate between different states of health.

**Table 1:** Phenotypic groups of participants included in the study

Gender	Age (years)	Group nr.	Phenotypic group	Body fat %	
Male	20–29	1	Reference group “optimal phenotypic flexibility”	Low to normal	< 20%
	30–59	2	Healthy range of phenotypes	Low	30–39 years < 8% 40–59 years < 11%
		3		Normal	30–39 years 8–20% 40–59 years 11–22%
		4		High	30–39 years > 20% 40–59 years > 22%
	60–70	5	Reference group “reduced phenotypic flexibility”	Normal to high	> 20%
Female	20–29	6	Reference group “optimal phenotypic flexibility”	Low to normal	< 30%
	30–59	7	Healthy range of phenotypes	Low	30–39 years < 21% 40–59 years < 23%
		8		Normal	30–39 years 21–33% 40–59 years 23–34%
		9		High	30–39 years > 33% 40–59 years > 34%
	60–70	10	Reference group “reduced phenotypic flexibility”	Normal to high	> 30%

### Design

All subjects were given the PhenFlex test (PFT) in the morning after an overnight fast (≥ 10 h). On study days before the first blood draw, a cannula was placed and blood samples were taken at *t* = 0 (fasting) and six time-points (*t* = 0.5, *t* = 1, *t* = 2, *t* = 4, *t* = 6, and *t* = 8 h) after consumption of the PFT. Subjects were not allowed to eat or drink until the last blood sampling, except from drinking water. Subjects were instructed to eat the same meal on the evening before the study day. Subjects were instructed to refrain from heavy physical activity/sports, alcohol, paracetamol, NSAIDs (i.e., ibuprofen, aspirin) starting 24 h before each study day.

### PhenFlex challenge

The 400 mL beverage consisted of a mixture of 12.40% (*w*/*w*) palm olein, 17.25% (*w*/*w*) dextrose, 4.13% (*w*/*w*) Protifar® (Nutricia), 0.10% (*w*/*w*) vanilla flavor, 0.12% (*w*/*w*) trisodium citrate, 0.08% (*w*/*w*) sodium hydroxide, and 66.12% (*w*/*w*) water. This resulted in a drink of 3950 kJ/950 kCal with a macronutrient composition of 60 g fat (of which 39% saturated fatty acids, 47% monounsaturated fatty acids, 14% polyunsaturated fatty acids), 75 g glucose, 5 g polysaccharides, and 20 g protein (analyzed by TNO Triskelion BV). The food-grade production of the beverage took place at the NIZO food research processing center in accordance with HACCP principles.

### Metabolic plasma parameters

Blood samples were collected in tubes containing clot activator for serum or in ice-chilled tubes containing Li-heparin or ethylenediaminetetraacetic acid (K_2_EDTA) as an anticoagulant for plasma and whole blood. In addition to K_2_EDTA, aprotinin was added to tubes for glucose-related parameters. After centrifugation (for 15 min at approximately 2000×*g* at approximately 4 °C within 30 min after collection), plasma and serum samples were stored at ≤ − 20 °C for clinical chemistry and ≤ − 70 °C for all other parameters. The following parameters were measured at 0 h (fasting) and six post-prandial time-points (0.5, 1, 2, 4, 6, and 8 h): clinical chemistry in serum—total cholesterol, HDL-cholesterol, LDL-cholesterol, triglycerides, nonesterified free fatty acids (NEFA), glucose, gamma-glutamyltransferase (GGT), ALAT, aspartate-aminotransferase (ASAT), alkaline phosphatase (ALP), albumin, and creatinine; glucose-related parameters—glucagon, insulin, and C-peptide in plasma by enzyme-linked immunosorbent assay (ELISA). Finally, GC metabolomics has been performed for the assessment of endogenous plasma metabolites by GCMS technology, where only a selection of a total of *n* = 26 amino acids and derivatives and ketone bodies were included according to the method described by Koek et al. [15]. All parameters were analyzed by TNO Triskelion BV.

### Indexes and summations

The Matsuda index was calculated according to Matsuda et al. (Eq. 1) [16].

1 $$ \frac{10000}{\sqrt{\left(\mathrm{fasting}\  \mathrm{insulin}\ \left(\mathrm{mU}/\mathrm{L}\right)\ \right)}\times \left(\mathrm{mean}\  \mathrm{insulin}\ \left(\mathrm{mU}/\mathrm{L}\right)\right)\times \left(\mathrm{mean}\  \mathrm{glucose}\ \left(\mathrm{mg}/\mathrm{dL}\right)\right)} $$

The hepatic insulin resistance index (HIRI) was calculated by the validated method of Matsuda et al. (Eq. 2).

2 $$ \mathrm{fasting}\  \mathrm{insulin}\ \left(\mathrm{mU}/\mathrm{L}\right)\times \mathrm{fasting}\  \mathrm{glucose}\ \left(\mathrm{mg}/\mathrm{dL}\right) $$

The concentration of non-essential amino acids was calculated as the sum of alanine, glutamine, glycine, proline, serine, and tyrosine. The concentration of aromatic amino acids (AAA) was calculated as the sum of phenylalanine, tyrosine, and tryptophan. The amount of branched-chain amino acids (BCAA) was calculated as the sum of isoleucine, leucine, and valine. Fisher’s ratio was defined as BCAA to AAA ratio [17]. We also calculated the phenylalanine hydroxylase activity index which is the concentration of tyrosine divided by the concentration of phenylalanine [18]. Finally, also the vitamin C index was calculated as concentration of proline divided by the concentration of hydroxyproline [19].

### Questionnaires

Apart from biomarkers derived from the blood, several questionnaires were administered: the State-Trait Anxiety Inventory (STAI), the short questionnaire to assess health-enhancing physical activity (SQUASH), and the food frequency questionnaire (FFQ) as well as screening questionnaires concerning disease, allergy, smoking, drug/alcohol, and sleep history and status, as well as education level and subjective evaluation of weight.

### Area under the curve calculations

For all parameters measured during the dietary challenge, incremental areas under or over the baseline were calculated using the first measurement (0 h) as a reference. The term area under the curve (AUC) refers to both values, which were delineated as negative AUC (AUC−) and positive AUC (AUC+).

### Questionnaire features

The inclusion of questionnaires in the health space model required conversion of answers into numerical values. Using each questionnaire question as a separate feature leads to sparse features; to prevent this, it was decided to summarize questionnaires to several constructed features. The feature “allergy” was composed of the questions: allergy for food or food components, allergy for iodine, allergy for latex, allergy for any medication, allergy for plasters, and any other allergy. The feature “disease” was composed of the questions to history of the following: cardiovascular disorder, gastrointestinal disorder, head/eyes/nose/throat disorder, hematological disorder, hepatic disorder, immunological disorder, endocrine/metabolic disorder, musculoskeletal disorder, neurological disorder, psychiatric disorder, pulmonary disorder, dermatological disorder, urogenital disorder, or any other disorder. This was done by setting the constructed feature value to 1 when any of the underlying questions reported positively and 0 when negative. Smoking, sleeping disorder, body weight perception, night-shift, and education level answers were converted to numerical values. Zero or 1 values were used for polar questions, while leveled answers were converted to − 1/0/1. Reported sleep hours were converted to numerical values. After the conversion, these questionnaire answers were used as features.

### Health space analysis

To evaluate PFT responses, a statistical visualization methodology called “health space” was applied, described earlier by Bouwman et al. [14]. In the current approach, we have adapted this methodology to use two reference groups to define the health space. This means that the health space was constructed to reflect differences between subjects from the reference group with “optimal phenotypic flexibility” and subjects from the reference group with “reduced phenotypic flexibility.” Having this predefined health space allowed for the visualization of individuals from “the healthy range of phenotypes,” so that their position in this space reflects their health status. The positioning of the healthy range of individuals in the health space is based on the regression values of the health space model, and since the health space is defined according to the difference between the two reference groups, the individuals’ health status is visualized on a spectrum ranging from one reference group to the other.

Axes were defined as four processes which characterize phenotypic flexibility, named (1) flexibility in glucose, (2) lipid, (3) amino acid and vitamin metabolism, and (4) metabolic stress. The first three axes represented metabolic components of phenotypic flexibility, while the fourth represented a measure of metabolic stress on the system as a whole. All measured parameters related to glucose metabolism and carbohydrate intake, lipid metabolism and fat intake, and amino acid metabolism and protein and vitamin intake were used for the axes named flexibility in glucose-, lipid-, amino acid and vitamin metabolism, respectively. The axis on metabolic stress was composed of parameters indicative for metabolic stress such as risk factors, injury markers, and well-being. Axes were not defined to be independent, since features were shared across axes. The metabolic stress axis had overlap with all other axes. The categories contain features from several types of data, namely clinical chemistry, metabolomics, anthropometry, several compound indices, and questionnaire answers. In order to utilize the data obtained during the challenge test in an interpretable way, the 0 h and the AUC− and AUC+ were used for plasma parameters. Subsequently, model performance and assessment of model overfitting with the selected features were evaluated according to the error rate. The error rate was defined as the misclassification rate of the two reference groups per axis of the health space model. For the model training, the “optimal phenotypic flexibility” reference group (aged 20–29, fat percentage low to normal) and the “reduced phenotypic flexibility” reference group (aged 60–70, fat percentage normal to high) were used. The model was trained to discriminate between these two reference groups using a separate tenfold double cross-validated (DCV) PLS-DA model for each of the four axes using their respective features. In this way, the model is repeated ten times in such a way that each individual has a 90% chance of being in the training or in the cross-validation set. In the DCV procedure, the model error was estimated independently of the model complexity and reported as a misclassification rate of the two reference groups per axis of the health space model and was calculated as the average percentage of individuals of the cross-validation set that is wrongly classified in each of the ten DCV-PLS-DA subtests. Since the health space model was composed of four different axes, four different error rates were being calculated. These error rates can be used to determine how relevant a biological process is for the separation of the two reference groups. In all analyses, all features were scaled to mean 0 and variance of 1. In every cross-validation step, the regression value for each parameter is saved (this equals the contribution of each biological compound to the model). Using these values, relative standard deviations (RSDs) of the regression value can be determined for each parameter. The order of magnitude of the RSD values gives an impression of the stability of the parameter for the importance of the discrimination between the reference groups in the axis and hence the relevance to the health space model. An instable parameter (having a high RSD value) may be of biological importance but acts as noise in the health space model. The RSD values were used as variable selection criterion. Parameters with high RSD values (200, 100, or 50%) were removed from the dataset, and the model was built again. In statistics, this approach of variable selection is called jackknifing.

All features used for each axis after jackknifing of the first health space are listed in Table 2. In the output of the health space model, the relative absolute regression vector values indicate relative importance of the respective feature on an axis.

**Table 2:** The selected features for the four axes of the health space

Glucose	Coefficients	Lipid	Coefficients	AA and Vit	Coefficients	Metabolic stress	Coefficients
C-peptide (AUCp)	0.062	LDL (t0)	0.060	D-Glutamic acid (t0)	0.054	LDL (t0)	0.060
Total carbohydrates (Q)	− 0.060	Body fat %	0.059	L-Tyrosine (t0)	0.040	Cholesterol (t0)	0.059
Polysaccharides (Q)	− 0.058	Cholesterol (t0)	0.059	L-Isoleucine (AUCp)	0.040	Systolic blood pressure	0.055
Glucose (t0)	0.055	Waist circumference	0.055	Vitamin C index	0.038	Current weight (Q)	0.046
4-Methyl-2-oxovaleric-acid (t0)	− 0.052	BMI	0.055	Fisher ratio	− 0.036	GGTP (t0)	0.041
3-Methyl-2-oxo-valeric-acid (t0)	− 0.049	Systolic blood pressure	0.054	Iron index	− 0.035	Diastolic blood pressure	0.041
Insulin (AUCp)	0.045	Diastolic blood pressure	0.040	L-Phenylalanine (AUCp)	0.035	Triglyceride (AUCp)	0.040
Matsuda index	− 0.045	Triglyceride (AUCp)	0.040	L-Leucine (AUCp)	0.035	Triglyceride (t0)	0.038
L-Isoleucine (AUCp)	0.045	Triglyceride (t0)	0.038	Albumin (t0)	− 0.034	Education (Q)	− 0.038
Mono- and disaccharides (Q)	− 0.044	Body weight	0.037	D-Glutamic acid (AUCn)	− 0.033	Glucose (t0)	0.037
Hepatic insulin resistance (HC)	0.041	Waist-hip ratio	0.037	Vitamin B6 (Q)	− 0.033	L-Tyrosine (t0)	0.031
Glucose (AUCp)	0.040	Triglyceride (AUCn)	0.028	Phenylalanine hydroxylase activity index	0.033	Matsuda index	− 0.031
L-Leucine (AUCp)	0.039	Total fat (Q)	− 0.023	AAA	0.032	Alcohol (Q)	− 0.029
3-Methyl-2-oxo-valeric-acid (AUCn)	0.032	3-Hydroxybutanoic acid (AUCp)	− 0.016	Vitamin B1 (Q)	− 0.031	Triglyceride (AUCn)	0.029
Glucagon (AUCp)	0.030			Albumin (AUCn)	0.029	Fisher ratio	− 0.028
L-Valine (AUCp)	0.029			L-Lysine (t0)	0.029	Glucose (AUCp)	0.027
4-Methyl-2-oxovaleric-acid (AUCn)	0.026			L-Methionine (t0)	− 0.027	L-Phenylalanine (AUCp)	0.027
Glucagon (AUCn)	0.024			L-Valine (AUCp)	0.026	Albumin (t0)	− 0.026
				Creatinine (AUCn)	0.025	Smoking (Q)	− 0.024
				L-Tyrosine (AUCp)	0.025	Allergy (Q)	− 0.024
				L-Ornithine (t0)	0.025	Albumin (AUCn)	0.023
				Vitamin B2 (Q)	− 0.024	Glucose (AUCn)	− 0.022
				Glycine (AUCp)	− 0.023	ALP (t0)	0.021
				L-Serine (AUCp)	− 0.022	L-Tyrosine (AUCp)	0.019
				Glycine (AUCn)	− 0.021	4-oxoproline (AUCp)	− 0.018
				Total protein (Q)	− 0.021		
				Folic acid (Q)	− 0.021		
				L-Valine (AUCn)	0.021		
				Threonine (AUCp)	− 0.020		
				Albumin (AUCp)	0.019		
				L-Leucine (AUCn)	0.018		
				L-Phenylalanine (AUCn)	0.018		
				L-Serine (t0)	− 0.018		
				L-Proline (AUCn)	0.016		
				L-Tyrosine (AUCn)	0.016		
				L-Asparagine (t0)	− 0.015		
				L-Asparagine (AUCp)	− 0.015		
				Nonessential AA	0.014		

Coefficients represent relative weights for the features, indicating importance for separation of reference groups. AUCp refers to positive part of AUC, AUCn to negative part of AUC, t0 to fasting measurements, and Q to questionnaire

GCMS-based features from the subjects in this study were combined with the same GCMS features from subjects of [6] in the second health space. Due to the combination of GCMS results, the two study outcomes had to be aligned for the creation of this health space, as this GCMS method produced relative concentrations. To do this, GCMS-based features were centered and normalized around a common value. Features from both groups were normalized separately around the means of their respective healthy controls. Because questionnaires were not available for the subjects in the second study, all questionnaire-related features had to be omitted in this instance. For the training of this model, the same reference groups were used as in the original health space.

After model training, all individuals were assigned values for each axis according to the regression vectors provided by the trained model. The resulting four values per individual represent coordinates on the four predefined axes of the health space model. The same eapproach was taken for the health space that combines data from two different studies. All axes are biologically and statistically interdependent and can be directly visualized in a 4D space. Model output includes error rates indicative of model performance.

### FlexScore

The FlexScore is a summarized ranking of subjects based on the preselected features. These preselected features were based on markers of which a higher or lower response to the PFT had a biological meaning in the sense that these could be interpreted as either beneficial or detrimental to health. Each subject was assigned a rank by summating all feature ranks for the subject. It is structured so that a higher score means less flexibility and vice versa. Additional file 1: Table S1 presents an overview of all features used for ranking.

For all features except Matsuda index, disposition index, and 3-hydroxybutanoic acid, rankings were made by ordering subjects from small to large values. For the remaining three features, subjects were ordered by values from large to small, as a small value for these features is associated with improved flexibility. This means that the FlexScore is a knowledge-based scoring system that provides an unbiased scoring of phenotypic flexibility, which was used to validate the health space model.

### Visualization of data

For the health space visualization, the axis scores of each individual were transformed into a JavaScript Object Notation (JSON) data structure. The JSON data format is a language-independent data exchange format specified by the RFC 7159 standard.

For the FlexScore visualization, the scores as well as the additional data for each subject were transformed into a JSON data structure as well. The JSON data structures were interpreted and manipulated for visualization using CanvasXPress (version 7.8, by Isaac Neuhaus, distributed under GNU GPLv3). CanvasXPress provides an interactive HTML5/JavaScript interface for easy data exploration and visualization. Correlations were visualized by CanvasXPress, Spearman’s rank correlation coefficients, and associated *p* values are provided by R (version 3.1.2 by the R Foundation for Statistical Computing, distributed under GNU GPLv3 with the Hmisc package, version 3.14-6 by Frank E. Harrell Jr.).

## Results

### Young and leaner subjects showed higher phenotypic flexibility in all health domains when compared to elderly subjects with higher adiposity

**Fig. 1:**
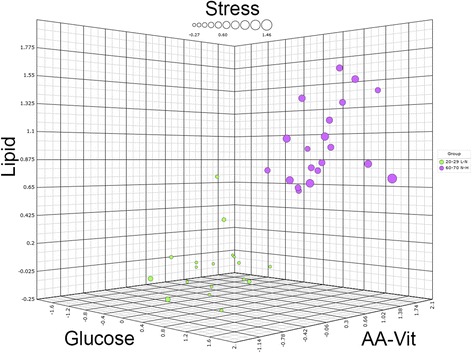
Overview of the reference groups in the main health space, with both sexes included. Three spatial axes are labeled for the domain they represent. “AA-Vit” stands for amino acids and vitamins. Groups labeled using their respective age intervals as well as their body fat percentage intervals, L for low, N for normal, and H for high. The dot size represents the “metabolic stress” axis of the health space

To investigate if male and female subjects of higher age (60–70 years of age) and normal to high body fat percentage would respond differently to PFT as compared to young subjects (20–30 years of age) with low to normal body fat percentage, we used the health space methodology. After training and optimizing the health space model using “optimal phenotypic flexibility” (20–29 years, low-normal fat percentage, see also Table 1) and “reduced phenotypic flexibility” (60–70 years, normal-high fat percentage, see also Table 1) reference groups, the two groups were seen to be well separated on all four axes of the health space (Fig. 1). The reduced phenotypic flexibility subjects center around a value of 1 for each of the axes, while the optimal phenotypic flexibility subjects center around 0. For the classification of the two reference groups used in this health space model error rates, which is the misclassification rate of the two reference groups per axis of the health space model after tenfold double cross-validation, were 13, 8, 23, and 3% for the glucose, lipid, amino acids and vitamin, and metabolic stress axes, respectively. After feature selection, 18 out of 46, 14 out of 32, 38 out of 75, and 25 out of 71 parameters, respectively, were important for separating the two reference groups for the glucose, lipid, amino acids and vitamin, and metabolic stress axes (Table 2).

For the axis representing flexibility in glucose metabolism, out of 18 included features, the positive AUC for C-peptide was the most important determinant for reference group separation, which increased in the “reduced phenotypic flexibility” group. Furthermore, results from the Dutch language FFQ were important for the glucose axis; total carbohydrates as well as polysaccharide intake were the second and third most important features for determining separation that decreased in the “reduced phenotypic flexibility” group. As the importance further declines, features which serve as common biomarkers in type 2 diabetes mellitus (T2D) appear (4-methyl-2-oxovaleric acid, 3-methyl-2-oxovaleric acid, fasting glucose, Matsuda index, and positive AUC for insulin) that all have higher concentrations in the “reduced phenotypic flexibility” group.

On the axis representing flexibility in lipid metabolism, fasting LDL and total fasting cholesterol were important determinants for reference group separation, as well as systolic and diastolic blood pressure, together with anthropomorphic features such as percentage body fat, waist circumference, and BMI. These are well-established clinical markers of metabolic syndrome as defined by the World Health Organization [20] and showed elevated concentrations in the “reduced phenotypic flexibility” as compared to the “optimal phenotypic flexibility” reference group.

The amino acid and vitamin flexibility axis used 38 features to create reference group separation, by far the most of any of the axes. While each of these features represents only a small contribution to the reference group separation, fasting glutamic acid is with some distance the most important feature. L-tyrosine, positive AUC of L-isoleucine, vitamin C index, and Fischer’s ratio are the following topmost important features. All features’ values are higher in the “reduced phenotypic flexibility” reference group, apart from Fischer’s ratio which is opposite.

The axis representing metabolic stress shared some of its most distinguishing features with the lipid axis. Here, fasting LDL, total cholesterol, and systolic blood pressure were the most important features together with self-reported satisfaction with body weight for separation of the two reference groups. Because these axes shared features, the behavior of the subjects along the lipid and metabolic stress axes is likely to be similar.

**Fig. 2:**
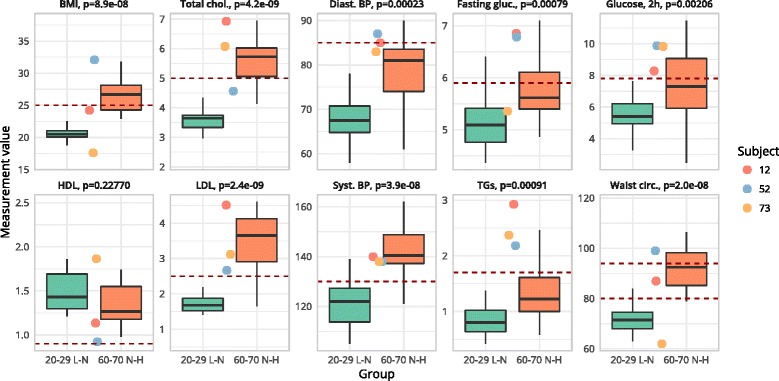
Boxplots for the two reference groups “optimal phenotypic flexibility” (green) and “reduced phenotypic flexibility” (red) as well as the values of the three outlier subjects (subjects 12, 52, and 73 shown in a red, blue, and yellow circle, respectively). Box shows the 25 to 75% interquartile range; whiskers indicate the maximum and minimum non-outlier values. Crossbar indicates the median values. Horizontal dotted line indicates cut-off values; levels above this line indicate abnormal clinical values. In waist, the lower dotted line represents cut-off values for female and the upper dotted line represents cut-off values for male; *p* indicates statistical significance after *t* test

To evaluate if the observed separation between the two reference groups with the health space methodology is reliable, different clinical markers (BMI and waist circumference; total, HDL, and LDL cholesterol; fasting glucose and 2 h glucose; systolic and diastolic blood pressure; and triglycerides) were being evaluated. All, except for HDL, were found to be significantly different (Fig. 2 and Table 3). In response to PFT persons of young age with low to normal fat percentage could be well discriminated from persons of higher age with normal to high fat percentage using the health space methodology.

**Table 3:** This table shows the mean (SD) values for the two reference groups “optimal phenotypic flexibility” (20–29 L to N) and “reduced phenotypic flexibility” (60–70 N to H) as well as the values of the three outlier subjects (subjects 12, 52, and 73)

Marker (unit)	20–29 L to N	60–70 N to H	Subject 12	Subject 52	Subject 73
BMI (kg/m2)^***^	20.61 (2)	27 (3.6)	24.2	32.1	17.6
Cholesterol (mmol/L)^***^	3.56 (0.5)	5.51 (0.9)	6.9	4.6	6.1
LDL (mmol/L)^***^	1.7 (0.4)	3.48 (0.8)	4.5	2.6	3.1
Glucose (mmol/L)^***^	5.11 (0.5)	5.73 (0.6)	6.8	6.8	5.4
HDL (mmol/L)	1.49 (0.2)	1.38 (0.3)	1.1	0.9	1.9
TG (mmol/L)^***^	0.82 (0.3)	1.43 (0.7)	2.9	2.2	2.4
Waist (cm)^***^	72.55 (7.7)	93.02 (10.1)	87	99	62
Sys. BP (mmHg)^***^	120.9 (9.1)	142.15 (10.4)	140	138	138
Dia. BP (mmHg)^***^	68.7 (6.5)	78.15 (8.1)	85	87	83
Glucose, 2 h (mmol/L)^***^	5.57 (1.2)	7.33 (2)	8.3	9.9	9.8

Indicated is the statistical significance level of the difference between the reference groups (***p < 0.005)

### Increasing body fat percentage is associated with a reduced resilience

**Fig. 3:**
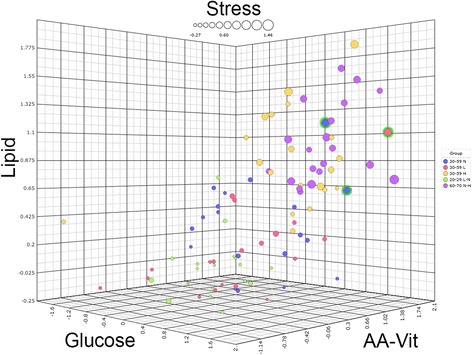
The main health space, with both sexes included. Three spatial axes are labeled for the domain they represent. “AA-Vit” stands for amino acids and vitamins. The dot size represents the “metabolic stress” axis of the health space. Groups labeled using their respective age intervals as well as their body fat percentage intervals, L for low, N for normal, and H for high. The three outlier subjects are encircled in green (subjects 12, 52, and 73)

To investigate if increasing adiposity (in terms of body fat percentage) would decrease phenotypic flexibility, we visualized male and female subjects from the “healthy range of phenotypes” in the health space model trained on the two reference groups. Figure 3 shows that most of the subjects in the age range of 30–59 years were distributed in between the “optimal” and “reduced phenotypic flexibility” reference groups on all four axes of the health space. From the pattern of distribution, we observed that subjects from the healthy range of phenotypes with a low fat percentage had partly overlapping and partly reduced resilience when comparing to the “optimal phenotypic flexibility” reference group. The subjects from the healthy range of phenotypes with a normal fat percentage had little overlap with the “optimal” nor “reduced phenotypic flexibility” reference groups and were located in the middle of the health space, in between the two reference groups. The subjects from the healthy range of phenotypes with a high fat percentage were considerably different as compared to the “optimal phenotypic flexibility” reference group and overlapped with the “reduced phenotypic flexibility” reference group who were of higher age. In response to PFT persons from the healthy range of phenotypes (aged 30–59 years) with increasing fat percentage showed reduced resilience using the health space methodology.

### Male type 2 diabetics showed reduced flexibility compared to healthy males, especially in the domains of glucose metabolism and metabolic stress

**Fig. 4:**
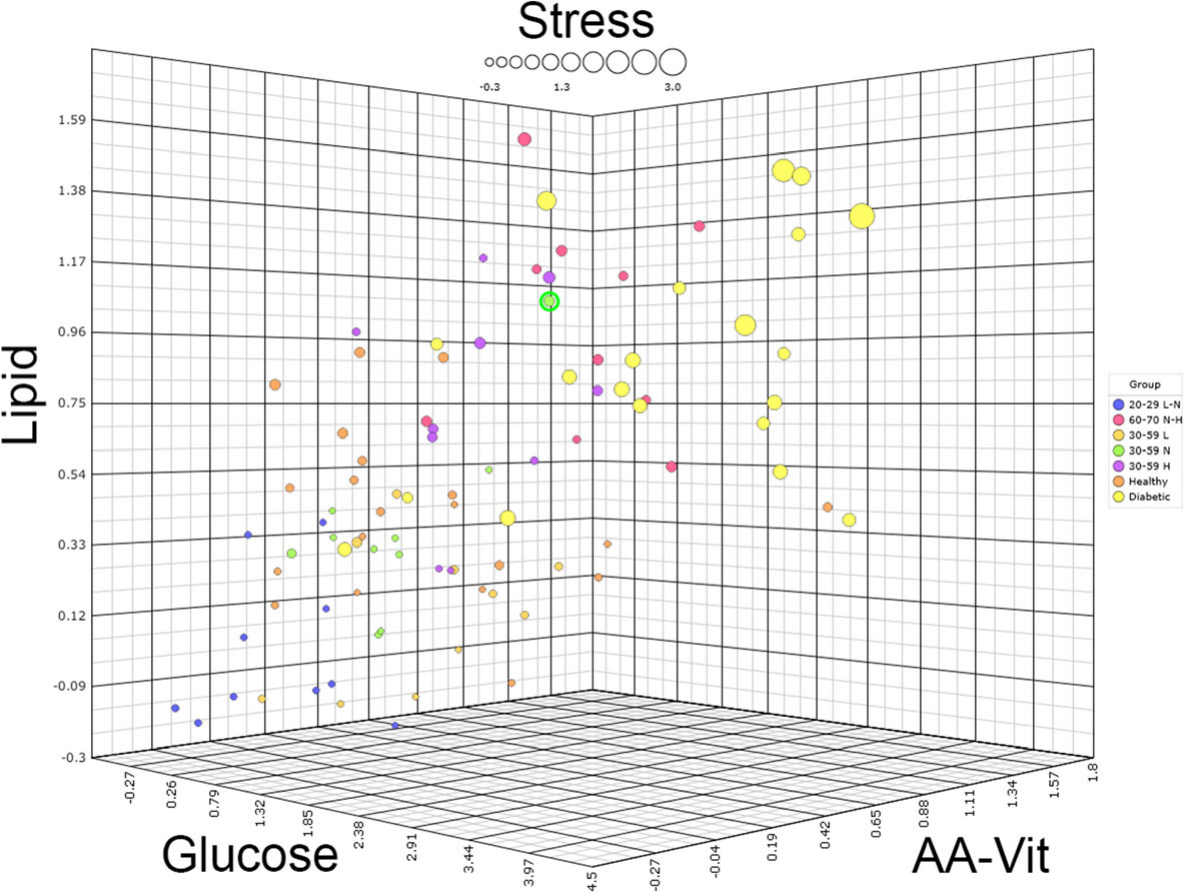
The combined health space including subjects from two different studies. Three spatial axes are labeled for the domain they represent. “AA-Vit” stands for amino acids and vitamins. The dot size represents the “metabolic stress” axis of the health space. Groups labeled using their respective age intervals as well as their body fat percentage intervals, L for low, N for normal, and H for high. The male outlier (subject 12) from the first health space is again encircled in green

To investigate if metabolically impaired subjects with type 2 diabetes could be discriminated from subjects with reduced phenotypic flexibility as well as from subjects from the “healthy range of phenotypes” with this health space model, data from male type 2 diabetics (T2D) were integrated with data from healthy males. Figure 4 shows a second health space which was created using the original male subjects from the healthy ranges study combined with data of subjects from a previous study using the same PFT and similar analytical platforms [6]. In this study, two groups were evaluated: male T2D and healthy controls. In order to project these subjects into a health space, only common features were used. After training and optimizing of the health space model using again males from the original two reference groups (“optimal” and “reduced phenotypic flexibility”), the maximum error rates were 10, 10, 15, and 0% for the glucose, lipid, amino acids and vitamin, and metabolic stress axes, respectively. Subsequently, this optimized health space model was used to visualize data from the healthy males and male T2D from the previous study. It was observed that the 20 healthy males (age range 30–55 with a mean average from 42 ± 7 and BMI range 20–25 with a mean average from 23 ± 1.5) from this previous study was positioned between the two reference groups, similar to the healthy range of male phenotypes with a normal fat percentage. It was clearly observed that 20 male T2D had a different position in the health space as compared to all groups from the healthy range of phenotypes, especially in the domain of glucose metabolism and metabolic stress. Together, these data showed that male T2D in response to PFT showed reduced flexibility compared to healthy males, especially in the domains of glucose metabolism and metabolic stress.

### FlexScore validates that health space model reflect individual phenotypic flexibility

To investigate if the health space model provides a biological valid representation of individual phenotypic flexibility as a measure of health, we wanted to test the individual health space representation against an independent and unbiased score of phenotypic flexibility. Therefore, a so-called FlexScore was calculated for the 100 subjects from the original study. The FlexScore was based on preselected features of which a higher or lower response to the PFT had a biological meaning in the sense that these could be interpreted as either beneficial or detrimental to health. A higher FlexScore indicated a lower overall phenotypic flexibility.

**Fig. 5:**
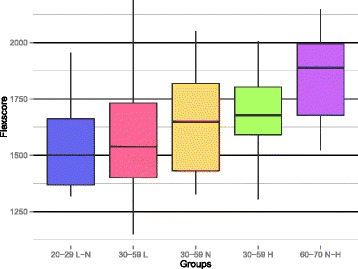
FlexScore distribution per study group. Box shows the interquartile range (IQR) with the median. The line indicates the range of observations within the ± 1.5 × IQR. Groups labeling used the respective age intervals as well as their body fat percentage intervals, L for low, N for normal, and H for high

When evaluating the FlexScores per phenotypic group (Table 1), it becomes apparent that a separation of groups was present in the FlexScores, similar to as what was observed in the health space model (Fig. 5). Again, the optimal phenotypic flexibility reference group (age 20–29 with a low to normal fat percentage) and the reduced phenotypic flexibility group (age 60–70 with a normal to high fat percentage) had the lowest and highest average FlexScores, respectively, (1539, SD = 199 and 1863, SD = 184). The three intermediate groups from the healthy range of phenotypes appeared each with an increasing FlexScore in proportion to their respective phenotypes (low fat percentage 1580, SD = 271; normal fat percentage 1655, SD = 248; high fat percentage 1696, SD = 175). The FlexScore showed a pattern that was remarkably similar to that observed in the health space. These FlexScore results substantiated, in an objective way, the findings from the health space.

### Phenotypic flexibility in conjunction with the health space is a reliable measure for individual health

To investigate how reliable the outcome of the health space model was as a measure for personalized health, we investigated the PFT biomarker response of the individual data of the so-called outlier subjects. Figure 3 shows outlier subjects (12, 52, and 73) that did not follow the general pattern seen for increasing age and body fat percentage. Figure 2 and Table 3 show the values for ten clinical parameters for the two reference groups as well as for these three outlier subjects.

Subject 52 is a female subject from the healthy range of phenotypes with a low body fat percentage that was visualized among subjects of the reference group with reduced phenotypic flexibility. This subject appeared to be the least flexible of all subjects in the amino acids and vitamin domain and among the least flexible for the other axes. A closer look at the biomarkers allowed for a clinical view of the health status for this particular individual when comparing her PFT responses to the two reference groups. The 59-year-old female subject had a high BMI and waist circumference despite a low fat percentage (subject 52, Figs. 2 and 3). This indicated the retention of fluids, perhaps in the form of edema. Fasting glucose, C-peptide, and ketones as well as glucose and glucagon PFT response indicated insulin resistance. Furthermore, the triglyceride and cholesterol PFT responses were above average when compared to the reference group with reduced phenotypic flexibility. Possible reduced liver functioning can be deduced by looking at Fischer’s ratio, which showed worse values as compared to the reference group with reduced phenotypic flexibility. More evidence for liver damage in this subject came from extreme PFT responses of ASAT and GGT, two features that were measured but not used in the construction of the health space model.

Also, two other subjects from the healthy range of phenotypes (subjects 12 and 73, Figs. 2 and 3) can be seen among the least flexible subjects which were not expected based by their age or fat percentage. Subject 12 was a 47-year-old male who showed a CVD risk phenotype with high total cholesterol and LDL, and high fasting triglycerides as well as elevated triglyceride PFT response, high ALP, and high GGT, despite a normal fat percentage and BMI when comparing his data to the reference group with reduced phenotypic flexibility. This clinical signature indicated possible liver steatosis. Furthermore, the data of this subject indicated impaired amino acid metabolism, shown by glutamic acid, tyrosine, methionine, and serine PFT response profiles. Furthermore, insulin sensitivity in this subject was decreased as indicated by fasting glucose, C-peptide, and Matsuda index as well as glucose and glucagon responses.

The third outlier, subject 73, was a 53-year-old male with a very low BMI and waist circumference. The response of the clinical parameters projected this individual among the subjects from the reference group with reduced phenotypic flexibility. His biomarker data indicated impaired lipid metabolism and visceral fattening. Elevated fasting cholesterol and triacylglycerol as well as triacylglycerol PFT response and diastolic blood pressure indicated disturbed lipid metabolism as do decreased 3-hydroxybutanoic acid and total ketone bodies. Liver steatosis is indicated by elevated ALP and the Fisher index. Furthermore, in this subject, glucose showed prediabetes, as indicated by glucose, C-peptide and glucagon responses, and the Matsuda index.

Interestingly, these three outlying subjects appeared to have extreme values for several non-clinical parameters, even when compared to T2D average values from data of a previous study [6]. All three subjects showed extreme values for 4-oxoproline (non-contributing). More specifically, subject 73 showed similarities to the T2D group for the triglyceride PFT response as well as for the ketone body feature. Subject 12 showed values more extreme than the average for the T2D group for fasting C-peptide, PFT glucose, and the PFT triglyceride response. For this same subject, the ASAT response is extreme and much higher as compared to the T2D group average. We refer to the supplementary for graphs showing several key parameters of these outlier subjects plotted against the values for the most flexible and least flexible reference groups. Based on the individual biomarker data of the three outlier subjects, it was shown that health space model outcomes provided a reliable measure for individual health.

## Discussion

In the current study, we showed that using the PFT and the analysis and visualization of predefined biomarker panels enabled the discrimination between different states of health. Within the healthy range of the population, it was possible to separate between subjects with optimal and suboptimal phenotypic flexibility since persons of young age with low to normal fat percentage had a markedly different position in the health space as compared to persons from old age with normal to high fat percentage in all four health domains which were glucose metabolism, lipid metabolism, amino acids and vitamins, and metabolic stress. This was also confirmed when evaluating a subset of clinical markers (BMI and waist circumference; total, HDL, and LDL cholesterol; fasting glucose and 2 h glucose; systolic and diastolic blood pressure; and triglycerides) that all showed significantly different values between the two reference groups except for HDL. Furthermore, it was shown that with increasing adiposity in terms of fat percentage, subjects decreased phenotypic flexibility. The health space including the 100 healthy subjects of both sexes showed that the individual phenotypic flexibility scores fall within a convex range, reaching from most flexible to least flexible. The general pattern within this data indicated a relationship between age and adiposity and the resulting phenotypic flexibility score on each of the four defined axes. This relationship between age, adiposity, and phenotypic flexibility was also observed in the unbiased FlexScore. In addition, the clinical marker subset for the three outlier subjects showed that multiple markers had values outside the normal range confirming their outlier position in the health space. Finally, this study showed that using the PFT and the analysis and visualization of predefined biomarker panels also enabled the discrimination between subjects from the healthy range of the population from diseased subjects with T2D. T2D male subjects had higher values for all of the four defined axes. In this health space, the glucose axis showed the largest overall range with [− 0.65 to 3.38] and [− 0.26 to 4.29] for healthy and T2D subjects, respectively. Perhaps surprisingly, of all axes, it was the metabolic stress axis with the largest range difference between groups; healthy subjects’ stress axis values range from − 0.3 to 1.53, while in the T2D group this ranges from 1.12 to 2.95. It can thus be concluded that these T2D subjects showed a reduced phenotypic flexibility in comparison with the most inflexible healthy individuals, even those of high age, especially for glucose metabolism and metabolic stress. Together, these results indicated that phenotypic flexibility as quantified by using the health space model appeared to be a reliable representation of individual health.

As a follow-up, the quantification of the metabolic PhenFlex test in conjunction with the health space methodology can be used to assess the effects of (nutritional) interventions on (individual) health. By applying PFT at various stages in the experiment and measuring and visualizing the biomarker responses according to the health space concept, one can assess health effects that occur by the (nutritional) intervention. In a parallel or crossover designed intervention study, the ideal behavior of the study groups would be that individuals from the control or placebo group occupy the same area of the health space, at the start and the end of the intervention period. The individuals from the intervention group would be expected to occupy the same area as would the control subjects at the start of the study, while they would move away from their starting position towards the “optimal health” reference group after the intervention period.

The aforementioned uses of the health space methodology concern group-wise comparisons. However, tracking changes for one individual across multiple occasions is one of the strengths of the health space approach. Because individuals are visualized within the health space in relation to the selected reference groups, it is possible to determine improvement or deterioration of individual health status, associated to time and/or treatment. The visual nature of the method enables intuitive display and tracking of individual health status. In personalized health, this can serve as both a personal feedback method and a way to determine and address areas of health in which changes are occurring, for targeted advice and interventions.

Several publications using a challenge test show that it has been difficult to show and interpret changes in health status as judged by changes in challenge-response induced by a nutritional intervention, especially for the less known nutrigenomics based biomarkers [2, 21, 22]. Our used approach would be able to facilitate accurate quantification of health changes as well as allow for intuitive interpretation of the results on a group and individual subject level simultaneously. This was shown for example by the description of the three outlier subjects. By putting their individual data in the context of PFT responses from the two reference groups, but also in the context of T2D responses, it was possible to provide an interpretation if the direction of the PFT biomarker response was beneficial or disadvantageous.

For one of the outlier subjects (subject 12), none of the features used for the generation of the health space appeared to be a true outlier. This means that an additive combination of features placed this subject among the least flexible. Without a composite-based biomarker model of health status, the highly decreased phenotypic flexibility of this subject would not be apparent. This subject is a prime example of the added value of the PFT biomarker response quantification in conjunction with health space visualization.

## Conclusions

The current study aimed to assess the ability of PFT to quantify flexibility in the healthy range of the population and whether it is possible to discriminate between optimal and suboptimal flexibility and therefore evaluated PFT response in 100 healthy male and female volunteers. We conclude that the results of the metabolic PFT in conjunction with the health space reliably assessed health on an individual basis. In response to PFT persons of young age with low to normal fat percentage could be well discriminated from persons of higher age with normal to high fat percentage using the health space methodology. Furthermore, in response to PFT persons from the healthy range of phenotypes (aged 30–59 years) with increasing fat percentage showed reduced resilience using the health space methodology. Finally, male T2D in response to PFT showed reduced flexibility compared to the full healthy range of males, especially in the domains of glucose metabolism and metabolic stress. The results from the work shown here may provide a toolbox for the quantification and interpretation of the effect of (nutritional) intervention studies on health status by quantifying phenotypic flexibility, be it in groups or for individuals. The use of this toolbox on an individual level opens up the possibilities for personal health diagnosis. Such a detailed and accurate personal health diagnosis can be used as a starting point for the generation of personalized health advices.

## Additional file

Additional file 1: Table S1. Parameters used to calculate the FlexScore. **Figure S1.** Upper figure shows the C-peptide response after the dietary challenge of the two reference groups together with those of the three outlier subjects (no. 12, 52, and 73). For the middle figure, this is the triglyceride response; the bottom figure shows the glucagon response. The red color shows the average values for the least flexible reference group (60–70 N-H) with the 95% confidence interval plotted around it. The green color shows the same for the most flexible reference group (20–30 L to N). (DOCX 372 kb)

## Abbreviations

AAA: Aromatic amino acids
ALAT: Alanine-aminotransferase
ALP: Alkaline phosphatase
ASAT: Aspartate-aminotransferase
AUC: Area under the curve
BCAA: Branched-chain amino acids
CCMO: Central Committee for Medical Research
CHDR: Centre for Human Drug Research
CVD: Cardiovascular disease
DCV: Tenfold double cross-validated
ELISA: Enzyme-linked immunosorbent assay
FFQ: Food frequency questionnaire
GCMS: Gas chromatography-mass spectrometry
GCP: Good clinical practice
GGT: Gamma-glutamyltransferase
HACCP: Hazard analysis and critical control point
HDL: High-density lipoprotein
HIRI: Hepatic insulin resistance index
JSON: JavaScript Object Notation
kCal: Kilocalorie
kJ: Kilojoule
LDL: Low-density lipoprotein
LUMC: Leiden University Medical Centre
NEFA: Nonesterified fatty acids
NSAID: Nonsteroidal anti-inflammatory drugs
PFT: PhenFlex test
RSD: Relative standard deviation
SQUASH: The short questionnaire to assess health-enhancing physical activity
STAI: The state-trait anxiety inventory
T2DM: Type 2 diabetes mellitus
w/w: Mass/mass
WMO: Wet medisch-wetenschappelijk onderzoek met mensen (Dutch medical research in Humans act)
Y: Year
