# Supplementary material for: Ranges of phenotypic flexibility in healthy subjects
Source: Genes Nutr. 2017 Dec 6;12:32. doi: 10.1186/s12263-017-0589-8 (PMC5718019; doi:10.1186/s12263-017-0589-8)
Supplement: Additional file 1: Table S1. — Parameters used to calculate the FlexScore. Figure S1. Upper figure shows the C-peptide response after the dietary challenge of the two reference groups together with those of the three outlier subjects (no. 12, 52, and 73). For the middle figure, this is the triglyceride response; the bottom figure shows the glucagon response. The red color shows the average values for the least flexible reference group (60–70 N-H) with the 95% confidence interval plotted around it. The green color shows the same for the most flexible reference group (20–30 L to N). (DOCX 372 kb) [file 12263_2017_589_MOESM1_ESM.docx]

**Additional file 1**

**Table S1.** Parameters used to calculate the flex score.

| \| Fasting Glucose \| Fasting ALAT \| \| --- \| --- \| \| AUCp Glucose \| AUCp ALAT \| \| AUCn Glucose \| AUCn ALAT \| \| Fasting triglycerides \| Fasting ASAT \| \| AUCp triglycerides \| AUCn ASAT \| \| AUCn triglycerides \| AUCn ASAT \| \| Fasting insulin \| Fasting ALP \| \| AUCp insulin \| AUCp ALP \| \| AUCn insulin \| AUCn ALP \| \| Fasting c-peptide \| Fasting total ketone bodies \| \| AUCn c-peptide \| Matsuda Index \| \| AUCn c-peptide \| Disposition Index \| \| Fasting glucagon \| Fasting 3-hydroxybutanoic acid \| \| AUCp glucagon \| AUCp 3-hydroxybutanoic acid \| \| AUCn glucagon \| AUCn 3-hydroxybutanoic acid \| \| Fasting free fatty acids \|  \| \| AUCp free fatty acids \|  \| \| AUCn free fatty acids \|  \| |  |
| --- | --- | --- | --- | --- | --- | --- | --- | --- | --- | --- | --- | --- | --- | --- | --- | --- | --- | --- | --- | --- | --- | --- | --- | --- | --- | --- | --- | --- | --- | --- | --- | --- | --- | --- | --- | --- | --- |

**Figure S1** Upper figure shows the C-peptide response after the dietary challenge of the two reference groups together with those of the three outlier subjects (no. 12, 52 and 73). For the middle figure this is the triglyceride response, the bottom figure shows the glucagon response. The red color shows the average values for the least flexible reference group (60-70 N-H) with the 95% confidence interval plotted around it. The green color shows the same for the most flexible reference group (20-30 L-N).
